# A laboratory test based on determination of cytokine profiles: a promising assay to identify exposition to contact allergens and predict the clinical outcome in occupational allergic contact dermatitis

**DOI:** 10.1186/s12865-015-0066-3

**Published:** 2015-02-06

**Authors:** Valentina Bordignon, Francesca Palamara, Giorgia Altomonte, Isabella Sperduti, Mario Pietravalle, Claudia Cavallotti, Paola Cordiali-Fei, Maria Pia Fuggetta, Antonio Cristaudo, Fabrizio Ensoli

**Affiliations:** Clinical Pathology and Microbiology, San Gallicano Dermatology Institute, Via Elio Chianesi 53, Rome, 00144 Italy; Allergy and Infectious Diseases, San Gallicano Dermatology Institute, Via Elio Chianesi 53, Rome, 00144 Italy; Epidermiology, Regina Elena Cancer Institute, Via Elio Chianesi 53, Rome, 00144 Italy; Clinical Dermatology, San Gallicano Dermatology Institute, Via Elio Chianesi 53, Rome, 00144 Italy; Institute of Translational Pharmacology (IFT), National Research Council (CNR), Rome, Italy

**Keywords:** Allergic contact dermatitis, Cytokines, ELISpot, Nickel, Occupation, Para-phenylenediamine, Patch test

## Abstract

**Background:**

Para-phenylenediamine (PPD) is the main allergen causing adverse reactions to hair dyes and a frequent cause of occupational-related skin sensitization among hairdressers and beauticians. The immunologic mechanism of the disease relies on the production of inflammatory cytokines by allergen-specific T cells, while regulatory T cells are thought to down-modulate the allergic response. This study was aimed at investigating the expression of effector or regulatory cytokines in exposed subjects in order to verify whether different cytokine profiles might predict distinct clinical outcomes. Peripheral blood mononuclear cells (PBMC) from 21 subjects occupationally exposed or not (10) to PPD were kept in short term cultures in the presence of optimized concentrations of NiSO4 × 6H2O or PPD. The production of IFN-γ and IL-10 elicited by antigens were analyzed by the ELISpot assay.

**Results:**

The presence of IFN-γ responses toward PPD was significantly correlated with a positive patch test (P = 0.002) and allergic symptoms, while IL10 responses were invariably found in PPD-exposed but clinically asymptomatic subjects with negative patch testing. We found concordance between the different cytokine profiles and patch test results. No false-positive results were found for the different cytokine profiles induced by PPD, resulting in 100% specificity. The sensitivity of the test was 87.5% (95% CI 65.9-100.0) with an overall test accuracy of 93.3%. Although larger prospective-retrospective studies are necessary to validate the predictive potential of the test, the negative and positive predicted values for PPD in this study were NPV = 87.5% and PPV = 100%, respectively.

**Conclusions:**

These data indicate that distinct cytokine profiles are associated with different clinical manifestations. The test, which is based on a simple and rapid profiling of cytokine responses by T lymphocytes against allergens, has proven to be a promising laboratory tool, useful for both the identification of previous contact with allergens and the etiologic diagnosis of contact allergies as well as capable of predicting the clinical outcome (development of an allergic or tolerant response).

## Background

Para-phenylenediamine (PPD) and related para-amino compounds represent the most common active reagents present in permanent dyes, which are widely used in a variety of industrial processes. PPD and related agents are potent contact allergens [1-5]. The relevance of PPD in the pathogenesis of delayed-type occupational skin allergy has increased considerably in recent years [6] and has been recognised as the main allergen causing severe adverse reactions to hair dyes [7,8]. In addition, PPD-related substances are also present in textile dyes [9], leather dyes [10], fur dyes, and ‘black’ rubbers [11]. Occupational sensitization to PPD and related substances presents the highest prevalence among hairdressers [12] and beauticians, in both men (15.4%) and women (14.7%) [5]. However, severe allergic reactions to PPD have been also reported in children [6]. In addition, the increasing use of permanent makeup or tattoos has recently become a further cause of PPD skin sensitization [13,14].

The clinical outcome of allergic contact dermatitis (ACD) includes contact urticaria, lymphomatoid reactions and even anaphylaxis [15], causing a considerable social and economic burden [7].

PPD-induced allergic disorders are mostly caused by skin contact to low molecular weight haptens. In fact, the effects of PPD on immune cells is thought to arise from its chemical instability under aqueous conditions. The ensuing auto-oxidation of the molecule leads to the formation of an electrophilic primary quinonediimine intermediate, which is susceptible to sequential self-conjugation. A rearrangement product of the oxido-conjugation reaction gives rise to the trimeric Bandrowski’s base, which is also immunogenic by itself [16,17].

The process of hypersensitivity in ACD requires a first sensitization phase, in which the hapten–carrier complex leads to T-cell activation [18-20] and is followed by the local release of pro-inflammatory cytokines. This causes skin inflammation with the involvement of keratinocytes, which is followed by epidermal changes, including spongiosis and, macroscopically, to the development of scales, vesicles or pustules [21]. Thus, immunologic mechanism responsible for ACD rely on the production of inflammatory cytokines by allergen-specific T cells, while regulatory cytokines are thought to down-modulate the allergic response. Previous studies have shown a relationship between the profile of cytokines induced by T cells and the presence of a skin reactivity to PPD, suggesting that IL-10 production exerts a “protective” effect while IFN-γ induces a “reactive” response [17,22,23]. These data support the hypothesis that a regulatory mechanism mediated by IL-10 contributes to the control of the clinical manifestations in response to allergens [24].

At present, diagnosis of ACD is based on clinical history and patch testing, the latter having major disadvantages, since interpretation of the results is subjective. Thus, the development of an accurate and reliable in vitro assay based on informative biomarkers and capable of predicting the clinical outcome, still represents an unmet need for the clinical and therapeutic management of ACD [25].

The present study was aimed at developing a laboratory test capable of exploring the cytokine profiles expressed by peripheral blood mononuclear cells (PBMC) in response to PPD and another common active allergen such as Nickel (Ni), and evaluating its specificity and sensitivity as well as the potential for predicting the clinical outcome in exposed subjects.

## Methods

### Patients

PBMC were obtained from 31 subjects. Twenty-one of them were hairdressers (13 women and 8 men). Eleven of them attended the Allergy outpatient clinic presenting moderate (n = 8) or severe (n = 3) symptoms of ACD, while 10 subjects were asymptomatic. Ten healthy subjects not occupationally exposed to PPD nor to Ni were studied as controls. The mean age was 33.2 yrs, with a range between 20–55. Subject’s description is summarized in Table 1.

**Table 1:** **Clinical details of patients**

**Patients**	**Age**	**Sex**	**Occupationally exposed**	**ACD symptoms**	**Patch test PPD**	**Patch test Ni**	**Other Patch test positivities**
1	25	f	Yes	Severe	+++	++	parabens, colophony, disp yellow
13	26	f	Yes	Severe	+++	++	No
2	25	f	Yes	Severe	++	neg	No
3	35	f	Yes	Moderate	++	neg	No
4	55	f	Yes	Moderate	++	neg	No
5	20	m	Yes	Moderate	+	neg	No
14	31	m	Yes	Moderate	++	neg	No
15	40	f	Yes	Moderate	+	neg	No
10	25	f	Yes	Moderate	neg	+	No
11	29	f	Yes	Moderate	neg	++	No
12	26	f	Yes	Moderate	neg	+	No
6	31	f	Yes	Asymptomatic	neg	neg	No
7	41	f	Yes	Asymptomatic	neg	neg	No
8	43	f	Yes	Asymptomatic	neg	neg	katon
9	33	m	Yes	Asymptomatic	neg	neg	No
20	41	f	Yes	Asymptomatic	neg	neg	No
21	41	m	Yes	Asymptomatic	neg	neg	No
22	33	m	Yes	Asymptomatic	neg	neg	No
23	35	m	Yes	Asymptomatic	neg	neg	No
24	32	m	Yes	Asymptomatic	neg	neg	No
25	33	m	Yes	Asymptomatic	neg	neg	No
26	35	f	Not	Asymptomatic	neg	neg	No
27	41	f	Not	Asymptomatic	neg	neg	No
28	43	f	Not	Asymptomatic	neg	neg	No
29	38	f	Not	Asymptomatic	neg	neg	No
30	38	f	Not	Asymptomatic	neg	neg	No
31	37	f	Not	Asymptomatic	neg	neg	No
32	25	f	Not	Asymptomatic	neg	neg	No
33	23	m	Not	Asymptomatic	neg	neg	No
34	24	m	Not	Asymptomatic	neg	neg	No
35	25	m	Not	Asymptomatic	neg	neg	No

Patient assessment was based on the administration of a questionnaire to collect information including occupational history, personal and familiar history of allergy (asthma and/or allergic rhino-conjunctivitis with at least one positive prick test reaction to relevant aeroallergens), and dermatitis. None of the patients had recently used immunosuppressive medication or underwent UV radiation or suffered from acute inflammatory skin diseases. Further, none of them had metal dental braces neither the presence of tattoo.

An informed consent was obtained from all subjects prior to the blood samples collection (Ethics Committee approval N. 488/14 IFO, Istituti Fisioterapici Ospitalieri).

### Patch testing

Patch testing was performed by Finn Chambers® on Scanpor® tape, with the European standard series of contact allergens (Hermal Trolab, Reinbeck, Germany), including 5% Nickel Sulphate hexahydrate (NiSO_4_ × 6H_2_O, Merck, AG, Darmstadt, F.R.G) and PPD 1% (FIRMA, Firenze, Italy; Chemotechnique Diagnostics, Vellinge, Sweden) applied in petrolatum. All allergens were applied on the upper back and removed after 48 hr.

Patch test responses were examined on day 2 and defined as strong (+++: oedema, erythema, papules and vesicles), moderate (++: oedema, erythema and papules), weak (+: oedema and erythema) or no reaction (neg) according to the International Contact Dermatitis Research Group guidelines [26].

### PBMC isolation

PBMC were isolated from 10 ml of heparinized blood, collected 48 hrs after skin testing, by standard Ficoll density-gradient centrifugation (Lympholyte-H solution Cederlane, Ontario, Canada) and washed twice with PBS. Cell aliquots were frozen in 90% heat inactivated Fetal Bovine Serum (FBS, Euroclone) and 10% DMSO (Dimethylsulphoxide, Sigma) and kept in liquid nitrogen.

### ELISpot determination of cytokine profiles induced in PBMC in response to allergens

Cytokine profiles were determined by Dual-Color Human ELISpot assay, according to manufacturer’s instruction. This test allow the simultaneous detection of IFN-γ and IL-10 secreting cells (R&D Systems, Europe Ltd., Abingdon, UK). Briefly, PBMC (3 × 10^5^ cells/well) from patients with positive patch test to PPD or Ni or both, or with negative patch test responses to either antigen, were incubated in triplicate (37°C, 5% CO_2_) for 48 h, in the presence or in the absence of the allergens, in RPMI-1640 medium containing 50 IU/mL penicillin, 50 μg/mL streptomycin, 2 mmol/L L-glutamine, 1× mixture of nonessential amino acids, 10% FCS (Gibco, BRL, UK). Red (IFN-γ) and blue (IL-10) spots generated by cells producing cytokines were assessed and recorded by an Automated ImmunoSpot Image Analyzer Software (AELVIS Technologies, TEMA ricerche, Italy).

The number of spot forming cells (SFC) per 3 × 10^5^ PBMC was used for the calculation of the Stimulation Index (S.I.), which express the ratio between stimulated and unstimulated cells [24]. Responses were considered positive when S.I. ≥3.

### Allergens

The allergens used for in vitro stimulation of PBMC were: Nickel sulphate hexahydrate NiSO_4_ × 6H_2_O, (Merck AG, Darmstadt, F.R.G), PPD (Sigma-Aldrich, Chemie, Kappelweg, Schnelldorf, Germany). Triplicate wells containing unstimulated cells or mitogen stimulated cells (PHA 1 μg/ml, Sigma, Saint Louis, Missouri, USA) were the negative and positive controls, respectively.

Stock solutions of PPD (1 M) were prepared dissolving the commercial powder in cell culture medium/DMSO (4:1, v/v) [27]. Preliminary titration experiments were performed with PPD on 5 different donors by testing different concentrations (0.1-1-10-20-40-50-100 μM) of PPD. The results were consistent with previous studies [27] and showed that concentration over 50 μM were toxic on PBMC (data not shown).

NiSO_4_ was resuspended before use, in sterile saline solution at 2 mg/ml (Bioindustria, Novi Ligure, Italy) and used at 20 μg/ml concentration as previously described [24].

All stock solutions were tested to exclude LPS contamination (Limulus assay, BioWhittaker, Cambrex Company, USA).

### Statistical analysis

Descriptive statistic was used to summarize pertinent study information. Comparisons between groups were performed for different variables using the non parametric Mann–Whitney test. Agreement between the in vitro results and clinical patch testing was estimated using the Cohen’s kappa test. Specificity, sensitivity, negative and positive predicted value (NPV and PPV, respectively) and accuracy were calculated. Significance was assessed at 5% level. All analyses were performed by SPSS for Windows statistical software (version 20; SPSS Inc., Chicago IL, USA).

## Results

### Patch testing

Patch test was performed in all 31 subjects with or without an history of occupational contact with PPD. Subject’s description and patch test results are detailed in Table 1. According to the skin response, 8 subjects out of 31 presented a skin response to PPD, while 5 subjects had responses to Ni. Two subjects (Id: 1 and 13, respectively) had positive responses to both allergens. All 20 asymptomatic subjects had negative patch test to PPD and Ni.

### Cytokine profiles in response to PPD and Ni

PBMC were incubated with PPD or Ni in short term cultures and IFN-γ and IL-10 production was measured by ELISpot assay. The results including SFC and S.I. for each subject are shown in Table 2. In all cases PHA stimulation (positive control) excluded individual differences in the capability of producing a specific cytokine as well as differences due to the quality of the cryopreservation.

**Table 2:** **Individual values of IFN-γ or IL-10 spot forming cells (expressed as mean values of triplicate wells) and S.I. for each experimental condition (W/O: medium alone; PHA: polyclonal stimulation 1 μg/ml; Ni 20 μg/ml, PPD 10 μM)**

	**IFN**							**IL-10**						
**Patients**	**W/O**	**PHA**	**PHA S.I.**	**Ni**	**Ni S.I.**	**PPD**	**PPD S.I.**	**W/O**	**PHA**	**PHA S.I.**	**Ni**	**Ni S.I.**	**PPD**	**PPD S.I.**
1	27.00	187.33	6.94	151.67	5.62	117.00	4.33	10.00	69.33	6.93	11.00	1.10	18.67	1.87
13	17.67	120.33	6.81	42.00	2.38	188.67	10.68	18.33	96.67	5.27	13.33	0.73	15.33	0.84
2	16.50	124.67	7.56	31.33	1.90	65.33	3.96	14.33	100.33	7.00	82.33	5.74	17.00	1.19
3	27.67	285.00	10.30	38.00	1.37	143.00	5.17	13.00	92.00	7.08	62.33	4.79	17.33	1.33
4	45.00	360.33	8.01	41.00	0.91	183.33	4.07	16.83	101.00	6.00	71.67	4.26	17.00	1.01
5	35.50	285.00	8.03	30.00	0.85	127.67	3.60	9.33	73.67	7.89	37.67	4.04	10.67	1.14
14	15.67	102.33	6.53	31.67	2.02	79.33	5.06	19.00	71.67	3.77	39.33	2.07	15.00	0.79
15	20.67	91.33	4.42	18.67	0.90	37.33	1.81	10.33	58.67	5.68	48.33	4.68	12.67	1.23
10	14.67	197.33	13.45	61.67	4.20	27.33	1.86	30.67	244.00	7.96	103.33	3.37	153.33	5.00
11	22.00	155.33	7.06	84.33	3.83	25.67	1.17	17.67	75.33	4.26	18.33	1.04	62.33	3.53
12	23.51	160.67	6.83	95.33	4.05	47.67	2.03	17.00	92.00	5.41	22.50	1.32	63.00	3.71
6	12.00	92.00	7.67	13.67	1.14	5.67	0.47	6.50	53.00	8.15	47.33	7.28	34.00	5.23
7	23.33	190.00	8.14	22.00	0.94	9.67	0.41	15.67	117.33	7.49	84.00	5.36	72.67	4.64
8	21.67	198.33	9.15	15.33	0.71	57.00	2.63	6.67	52.00	7.80	32.00	4.80	11.33	1.70
9	10.33	70.33	6.81	9.67	0.94	16.33	1.58	32.67	273.00	8.36	133.67	4.09	170.33	5.21
20	17.00	72.33	4.25	18.67	1.10	10.67	0.63	4.50	57.00	12.67	42.67	9.48	37.33	8.30
21	7.33	204.67	27.91	8.33	1.14	15.00	2.05	16.00	104.67	6.54	88.33	5.52	76.00	4.75
22	26.00	195.67	7.53	35.33	1.36	59.67	2.29	10.33	47.00	4.55	37.33	3.61	32.33	3.13
23	14.00	72.00	5.14	12.00	0.86	18.33	1.31	31.67	277.00	8.75	139.33	4.40	173.67	5.48
24	10.00	97.00	9.70	17.00	1.70	8.33	0.83	9.83	54.00	5.49	50.67	5.15	36.00	3.66
25	17.67	207.33	11.74	26.67	1.51	13.00	0.74	19.33	121.00	6.26	86.00	4.45	76.00	3.93
26	18.33	208.67	11.38	18.67	1.02	27.33	1.49	7.00	57.33	8.19	16.67	2.38	11.33	1.62
27	3.67	70.33	19.18	9.67	2.64	9.00	2.45	32.67	273.00	8.36	33.67	1.03	70.33	2.15
28	21.67	228.67	10.55	22.00	1.02	30.67	1.42	4.00	56.67	14.17	11.67	2.92	9.33	2.33
29	32.67	68.33	2.09	13.00	0.40	16.00	0.49	3.67	43.67	11.91	9.67	2.64	9.00	2.45
30	20.00	206.00	10.30	19.33	0.97	33.33	1.67	9.67	52.00	5.38	28.00	2.90	14.67	1.52
31	25.00	66.33	2.65	26.33	1.05	23.00	0.92	32.67	173.00	5.30	32.00	0.98	66.00	2.02
32	6.00	36.00	6.00	15.33	2.56	17.67	2.94	6.67	52.00	7.80	15.33	2.30	11.33	1.70
33	9.33	51.00	5.46	9.67	1.04	16.33	1.75	28.33	212.33	7.49	50.00	1.76	62.67	2.21
34	32.00	208.67	6.52	19.33	0.60	29.00	0.91	7.33	53.00	7.23	18.67	2.55	12.00	1.64
35	5.33	70.33	13.19	6.33	1.19	14.33	2.69	21.67	273.00	12.60	26.67	1.23	27.00	1.25

**Figure 1:**
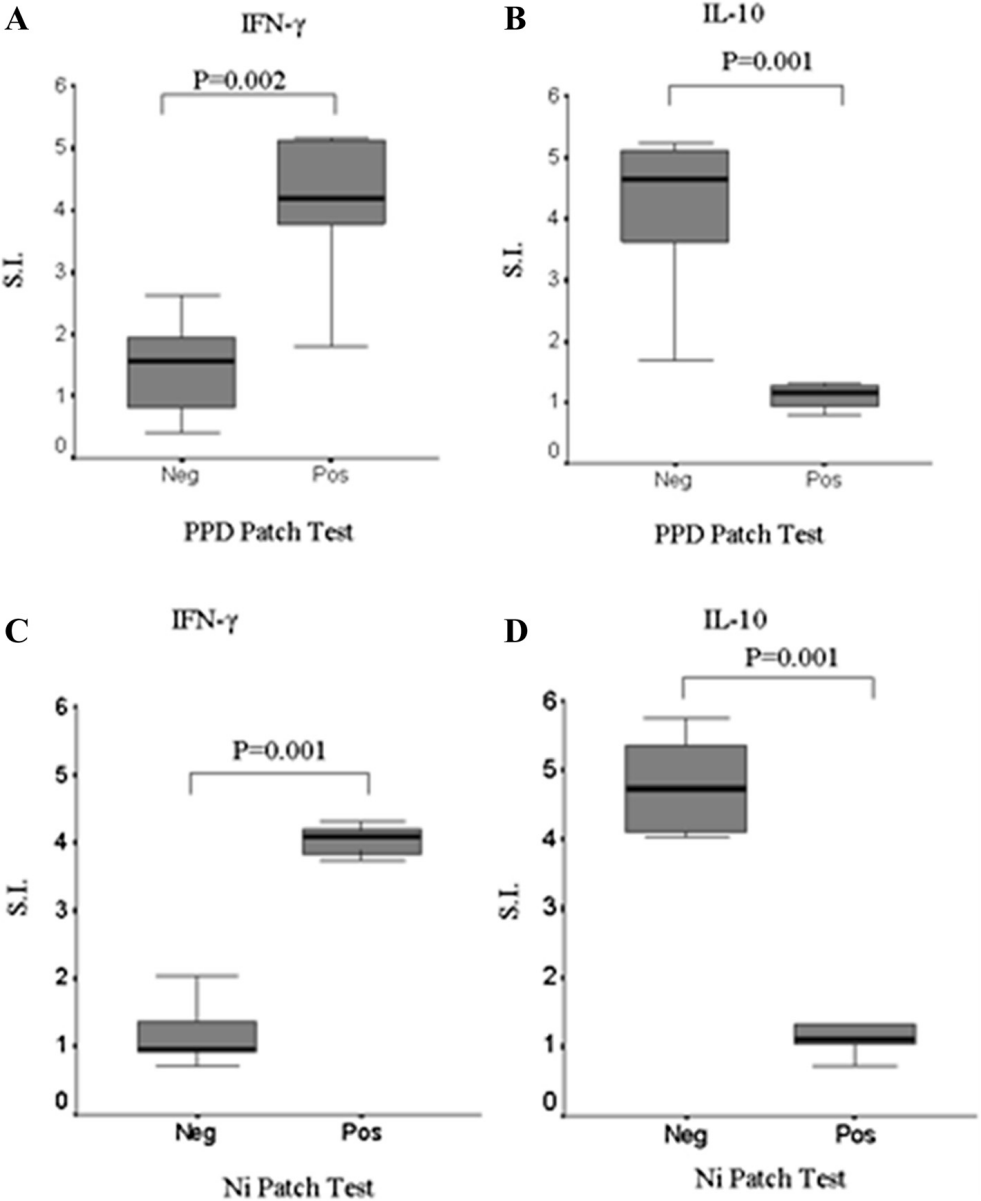
**Cytokine response elicited by PPD (panel A and B) or Ni (panel C and D) by ELISpot analysis in patch test negative or positive patients, respectively.** ELISpot results of detection of IFN-γ (panel **A-C**) or IL-10 (panel **B-D**) producing PBMC upon stimulation with Ni (20 μg/ml) or PPD (10 μM) were expressed as the mean values of S.I. ± SD. S.I. Index value is expressed by the ratio between the number of spot forming cells (IFN-γ and IL-10 producing PBMC) upon stimulation with the allergen and those present in the absence of stimuli (spontaneous cytokine production). Positive IFN-γ-responses (Index ≥ 3), in the presence of PPD or Ni, were detected only in subjects with positive patch test to PPD (panel **A**, P = 0.002) or to Ni (panel **C**, P = 0.001), respectively.

The production of IFN-γ was strictly associated with a positive patch test result (Table 3). In fact, Positive IFN-γ-responses (Index ≥ 3), in the presence of PPD or Ni, were detected only in subjects with positive patch test to PPD (Figure 1, panel A, P = 0.002) or to Ni (Figure 1, panel C, P = 0.001), respectively.

**Table 3:** **PPD or Ni patch test results and respective S.I. IFN-γ values in response to antigens**

**Patients**	**Patch test PPD**	**SI PPD**	**Patch test Ni**	**SI ni**
1	+++	**4.33**	++	**5.62**
13	+++	**10.68**	++	**2.38**
2	++	**3.96**	neg	**1.90**
3	++	**5.17**	neg	**1.37**
4	++	**4.07**	neg	**0.91**
5	+	**3.60**	neg	**0.85**
14	++	**5.06**	neg	**2.02**
15	+	**1.81**	neg	**0.90**
10	neg	**1.86**	+	**4.20**
11	neg	**1.17**	++	**3.83**
12	neg	**2.03**	+	**4.05**
6	neg	**0.47**	neg	**1.14**
7	neg	**0.41**	neg	**0.94**
8	neg	**2.63**	neg	**0.71**
9	neg	**1.58**	neg	**0.94**
20	neg	**0.63**	neg	**1.10**
21	neg	**2.05**	neg	**1.14**
22	neg	**2.29**	neg	**1.36**
23	neg	**1.31**	neg	**0.86**
24	neg	**0.83**	neg	**1.70**
25	neg	**0.74**	neg	**1.51**
26	neg	**1.49**	neg	**1.02**
27	neg	**2.45**	neg	**2.64**
28	neg	**1.42**	neg	**1.02**
29	neg	**0.49**	neg	**0.40**
30	neg	**1.67**	neg	**0.97**
31	neg	**0.92**	neg	**1.05**
32	neg	**2.94**	neg	**2.56**
33	neg	**1.75**	neg	**1.04**
34	neg	**0.91**	neg	**0.60**
35	neg	**2.69**	neg	**1.19**

All asymptomatic subjects with a history of contact with PPD, however presenting a negative patch test, had invariably an IL-10 response in the absence of IFN-γ production. Healthy controls did not produce IFN-γ nor IL-10 in response to stimulation with PPD or Ni.

As shown in Figure 1, IFN-γ or IL-10 production in response to allergens appear mutually exclusive, while PHA is capable of inducing both cytokines (Table 2). Statistical analysis confirmed a significant negative correlation between IFN-γ and IL-10 production (P < 0.0001).

No false-positive test results were found for PPD- neither for Ni-induced increments, thus giving a 100% specificity for both allergens. On the other hand, the sensitivity was calculated as 87.5% (95% CI 65.9-100.0) for PPD and 80% (95% CI 57.4-100.0) for Ni, respectively, hence the overall test accuracy was of 93.3%.

A satisfactory concordance agreement between in vitro and patch test results was also found (Cohen’s kappa test 0.84, P = 0.001).

The negative and positive predicted values were NPV = 87.5% and PPV = 100% for PPD and NPV = 90.9% and PPV = 100%, for Ni, respectively.

## Discussion

This study was aimed at exploring the cytokine responses to specific allergens to evaluate whether the analysis of different cytokine profiles might provide the basis for a laboratory test capable of identify an allergic sensitization.

To date, the patch test is considered the gold standard for identifying the causative agents responsible for contact allergy [28]. However, its use is currently debated [4,29]. In fact, although having a high sensitivity, patch test has a major disadvantage, since interpretation of the results is subjective. In addition it might be the cause of a iatrogenic sensitization and, although rarely, induce adverse reactions [30]. Thought desirable, a laboratory test capable of supporting the clinical and therapeutic management of ACD it is not available, as yet.

Evidence indicate that the clinical manifestations of ACD are associated with an inflammatory response to allergens [22] while “regulatory” responses [21] are found in allergen-responsive subject, which however do not develop clinical symptoms. In fact, previous studies [24,31,32] suggested that the production of specific cytokines in response to antigenic stimulation can effectively modulate the type of immune response. In particular, this paradigma strongly suggests that IFN-γ production is associated with a “reactive” phenotype, which gives rise to clinical symptoms, while IL-10 production exerts a “protective” effect, capable of controlling the hypersensitivity symptoms in response to allergens [33]. This notion prompted us at exploring the pattern of cytokine expression elicited “in vitro” by contact allergens for the development of a novel, simple, robust and reliable laboratory test for the diagnosis and clinical profiling of ACD patients.

Other laboratory test, including the lymphocyte activation test (LAT) and lymphocyte transformation test (LTT), have been proposed to investigate drug and contact allergy [34-37]. These test are based on the detection of antigen-driven T cell proliferation by [^3^H]-thymidine incorporation. However these approaches have shown a low reproducibility, being therefore difficult to standardize, and require radioactive compounds. Furthermore they do not provide any predictive information about the potential clinical outcome.

The present study was based on the assessment of the cytokine profiles, namely IFN-γ and IL-10 production by T cells, in response to allergens, using a Dual-Color ELISpot assay [38,39].

We found a significant correlation between the ELISpot results and the results gathered by patch testing (P = 0.001). In particular, IFN-γ responses against PPD or Ni were found only in subjects with a positive patch test to PPD (P = 0.002) or to Ni (P = 0.001), respectively. On the contrary, all clinically asymptomatic subjects, with a history of regular contact with PPD, had a negative patch test but showed an IL-10 production in response to the allergens, invariably in the absence of IFN-γ production, while, control healthy subjects did not produce IFN- γ nor IL-10. These data confirm the presence of an anamnestic, though clinically asymptomatic, response to the allergen even in exposed subject which present with a negative patch test. In fact, IFN-γ or IL-10 production appear mutually exclusive, as confirmed by the statistical treatment of the data. The assay gave a 100% specificity, a 80–87,5% sensitivity and a 93.3% accuracy.

## Conclusions

Although larger prospective-retrospective studies are necessary to validate the predictive potential of the test and the possible indications for its use in clinical practice, the results suggest that this assay might offer a complementary or, in some cases, alternative diagnostic tool for the assessment of individuals with known or suspected exposure to cutaneous allergens, particularly in those cases of difficult application of patch test as in patients with chronic skin inflammation, while helping avoid the risk of both subjective interpretation and iatrogen sensibilization. In addition, it may be useful to reveal previous exposure to allergens both in asymptomatic and “allergic” individuals and might prove effective at predicting the clinical outcome as well as at monitoring the effect of therapeutic regimens.

## Abbreviations

PPD: Para-phenylenediamine
Ni: Nickel
PBMC: Peripheral blood mononuclear cells
SFC: Spot forming cells
S.I.: Stimulation index
LAT: Lymphocyte activation test
LTT: Lymphocyte transformation test
